# Case Report: Signal Transducer and Activator of Transcription 3 Gain-of-Function and Spectrin Deficiency: A Life-Threatening Case of Severe Hemolytic Anemia

**DOI:** 10.3389/fimmu.2020.620046

**Published:** 2021-01-15

**Authors:** Sara Ciullini Mannurita, Rayan Goda, Ebe Schiavo, Maria Luisa Coniglio, Annachiara Azzali, Ilaria Fotzi, Annalisa Tondo, Veronica Tintori, Stefano Frenos, Maria Chiara Sanvito, Marina Vignoli, Cristina Luceri, Elisabetta Bigagli, Alessia Grassi, Mario Milco D’Elios, Claudio Favre, Eleonora Gambineri

**Affiliations:** ^1^ Division of Pediatric Oncology/Hematology, Meyer University Children’s Hospital, Florence, Italy; ^2^ Department of Neurosciences, Psychology, Drug Research and Child Health (NEUROFARBA), University of Florence, Florence, Italy; ^3^ Department of Experimental and Clinical Medicine, University of Florence, Florence, Italy

**Keywords:** STAT3 gain-of-function, spectrin deficiency, primary immune regulatory disorders, hereditary spherocytosis, hemolytic anemia, autoimmune lymphoproliferative syndrome, lymphadenopathy, hematopoietic stem cell transplantation

## Abstract

*STAT3* gain-of-function (GOF) mutations can be responsible for an incomplete phenotype mainly characterized by hematological autoimmunity, even in the absence of other organ autoimmunity, growth impairment, or severe infections. We hereby report a case with an incomplete form of *STAT3* GOF intensified by a concomitant hereditary hematological disease, which misleads the diagnosis. The patient presented with lymphadenopathy, splenomegaly, hypogammaglobulinemia, and severe autoimmune hemolytic anemia (AIHA) with critical complications, including stroke. A Primary Immune Regulatory Disorders (PIRD) was suspected, and molecular analysis revealed a *de novo STAT3* gain-of-function mutation. The response to multiple immune suppressive treatments was ineffective, and further investigations revealed a spectrin deficiency. Ultimately, hematopoietic stem cell transplantation from a matched unrelated donor was able to cure the patient. Our case shows an atypical presentation of *STAT3* GOF associated with hereditary spherocytosis, and how achievement of a good long-term outcome depends on a strict clinical and laboratory monitoring, as well as on prompt therapeutic intervention.

## Introduction

*STAT3* gain-of function (GOF) germline mutations cause a Primary Immune Regulatory Disorders (PIRD) characterized by multisystem autoimmune diseases (e.g. autoimmune thyroid disease, enteropathy, diabetes, arthritis and interstitial lung disease), hematologic manifestations (autoimmune cytopenias, hypogammaglobulinemia, lymphopenia), increased susceptibility to infections and growth delay/failure to thrive (1). The disease clinical presentation is heterogeneous as there is no obvious genotype/phenotype correlation, and a differential diagnosis with other causes of Immune Regulatory Disorders may be difficult to achieve. The enhanced STAT3 activity reflects on an impaired cytokine signaling and regulation of other STAT molecules, high levels of IL-6, decreased number and function of T regulatory cells and increased numbers of T helper 17 cells (2). Autoimmune cytopenias are included in this disease spectrum, in particular autoimmune hemolytic anemia (AIHA) (1, 2). Nevertheless, hemolytic anemia can also be caused by hereditary red cell membrane disorders, such as spectrin deficiency, which is one of the most common causes of hereditary spherocytosis (3). Jaundice, hemolytic anemia, splenomegaly and gallstone formation are associated clinical features, and display high variability between mild and more severe forms described (3, 4). In this report, we present a unique case of severe refractory hemolytic anemia, with life-threatening complications due to *STAT3* GOF mutation aggravated by spectrin deficiency.

## Methods 

### Immunophenotyping

Flow cytometric analysis was performed on ethylenediaminetetraacetic acid (EDTA) blood samples processed within less than 24 h after collection. Red blood cells were lysed with ammonium chloride and lymphocytes were stained to identify T and B cell subsets and NK cells using the following monoclonal antibodies: CD45 APC-H7 or CD45 VioGreen (Miltenyi Biotec), CD3 VioGreen (Milteny Biotec), CD8 VioBlue (Miltenyi Biotec), CD4 PerCP-Cy5.5, CD19 PerCP-Vio 700 or CD19 PE, CD56 VioBright 515 (Miltenyi Biotec), CD31 APC, CD27 PE or CD27 APC, TCRαβ APC, TCRγδ PE, HLA-DR APC, IgM FITC, IgD PE. All antibodies were purchased from BD Biosciences unless otherwise noted.

Cells were stained for 15 min at room temperature, washed with PBS and resuspended in PBS. Flow cytometry data were collected using a MACSQuant Analyzer 10 flow cytometer (Miltenyi Biotec) and analyzed with Flowlogic Software (Inivai). CD3, CD4, CD8, CD27, CD45RA, CD31 were used to identify naïve (CD27+CD45RA+), central memory (CD27+CD45RA-), effector memory (CD27-CD45RA-), terminally differentiated effector memory T cells (CD27-CD45RA+) and recent thymic emigrants (CD45+CD31+). CD25 and CD127 allow the identification of Treg in CD4 population (CD25+CD127low). Double negative T cells were identified based on the expression of TCRαβ in CD4-CD8- T subpopulation (TCRαβ+CD4-CD8-). Activated T cells were characterized by the expression of HLA-DR and CD3 markers.

CD19+ B cell subsets were defined based on the differential expression of CD27 and IgD into naïve (CD27-IgD+), marginal zone-like (CD27+IgD+), class switch (CD27+IgD-). NK cell populations were defined based on the expression of CD56 (CD3-CD56+).

Absolute cells count was calculated from total lymphocyte numbers obtained by differential blood count (A. Meyer Children Hospital, Florence, Italy).

### Next Generation Sequencing (NGS) Analysis

Genomic DNA (gDNA) was isolated from peripheral blood using the QIAamp DNA Blood Mini Kit (Qiagen) and quantified. Sequencing was performed using the MiSeq Illumina platform (Illumina), according to the protocols indicated. Sequence reads were aligned to the NCBI38/hg38 reference genome using a pipeline based on BWA and variants were called using the GATK toolkit. Variants annotation and prioritization was performed according to an in-house developed pipeline, on a selected genes panel (**Table S2**). Variants pathogenicity was evaluated, according to the standard and guidelines of the American College of Medical Genetics and Genomics (ACMG) (5), by using a combination of prediction programs (SIFT, PolyPhen, pMUT, Mutation taster, FATHMM score, CADD score) to distinguish potentially damaging variants from those predicted to have neutral effect. NGS results have been confirmed by Sanger analysis.

### Chimerism Analysis

Chimerism was evaluated by multiple fluorescent short tandem repeat analysis using AmpFlSTR Identifiler Plus PCR amplification kit (Thermo Fisher Scientific), according to the manufacturer’s instructions. The tetranucleotide STR loci amplified in this reaction included: D8S1179, D21S11, D7S820, and CSF1PO (all labeled with 6-FAM blue dye); D3S1358, TH01, D13S317, D16S539, and D2S1338 (all labeled with VIC green dye); D19S433, vWA, TPOX, and D18S51 (all labeled with NED yellow dye); and D5S818 and FGA (all labeled with PET red dye). In addition, the amelogenin locus was analyzed to differentiate X and Y chromosome (labeled with PET red dye). The PCR products were analyzed using a 3500 Genetic Analyzer (Thermo Fisher Scientific). Fragment size and peak area data were determined by GeneMapper software (Thermo Fisher Scientific). The degree of chimerism was calculated using the method described elsewhere (6).

### CYP3A4 SNP Genotyping

PCR-Restriction Fragment Length Polymorphism (PCR-RFLP) genotyping was performed on HC and patient’s genomic DNA (gDNA) as previously described (7). Briefly, HC and patient’s gDNA was isolated and amplified using primers specific for CYP3A and CYP3A4*1G sequences. PCR products were then digested with RsaI restriction enzyme and analyzed by electrophoresis on a 3% agarose gel. RsaI digestion of wild-type DNA yields 217 bp and 70 bp fragments whereas, in presence of CYP3A4*1 variant allele, it yields a 287 bp band. Results were confirmed by direct sequencing.

### Cytokine Production

The cytokine serum levels were measured by multiple bead-based immunoassay according to the manufacturer’s protocol (Thermo Fisher Scientific).

## Case Description

**Figure 1:**
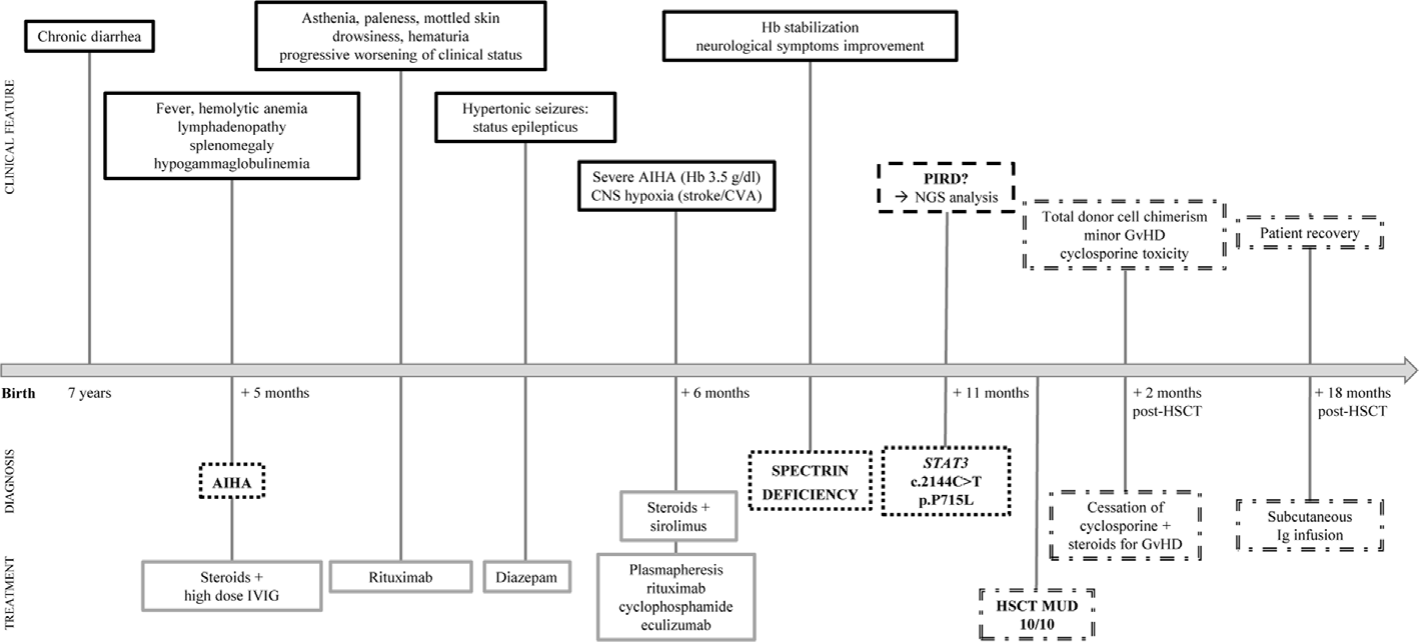
Timeline of patient’s clinical presentation, diagnosis and treatment. Clinical features (solid black lines), diagnosis (black dotted lines), treatment (solid gray lines), hypothesis (dashed black lines) and hematopoietic stem cell transplantation (HSCT) outcome (double dashed lines) are indicated.

We here describe the case of an 11-year old girl, coming to our attention aged 8 due to lymphadenopathy and anemia. The patient was the second child of non-consanguineous parents with no family history of hematological diseases. She was healthy until the age of 7, when she suffered from recurrent episodes of diarrhea, lasting approximately 6 months, for which the investigations performed did not lead to any relevant diagnosis. Few months later she presented with generalized lymphadenopathy without any evidence of infectious or malignant etiology. At time of referral the patient complained of fever, asthenia and lymphadenopathy. Her examination revealed multiple enlarged lymph nodes and splenomegaly. Laboratory investigations showed a significantly low hemoglobin (Hb) levels (**Table 1**). Direct Coombs tests was positive, thus indicating an immune-mediated hemolytic anemia. Moreover, mild hypogammaglobulinemia was noted, with decreased CD3^+^ and CD8^+^ lymphocytes and raised α/β double negative T cell (DNT) population (**Table 1**), strengthening the hypothesis of an immune mechanism responsible for the clinical manifestations. This was further confirmed by reactive changes, with follicular hyperplasia at axillary lymph node biopsy. Autoimmune lymphoproliferative Syndrome (ALPS) was suspected, and the patient was treated with prednisolone (2 mg/kg/day), with good response and increase of Hb levels to 11.2 g/dl. She also underwent intravenous immunoglobulin (IVIG) replacement therapy for hypogammaglobulinemia. However, one week later, she presented with extreme pallor, fatigue and severe anemia with hemoglobin levels of 4.4 g/dl and strong positive direct Coombs test. Her condition deteriorated, and she developed generalized convulsions with decreased level of consciousness. Hemoglobin levels further dropped to 3.5 g/dl and she was admitted to the pediatric intensive care unit. Brain computed tomography showed multiple ischemic lesions in the putamen area. The patient received plasmapheresis and subsequently rituximab, cyclophosphamide and eculizumab as second-line therapy to control the severe hemolysis. Meanwhile, sirolimus was started as immune modulator. She achieved a good clinical response and corticosteroids and sirolimus treatment was maintained. The patient also started a specific rehabilitation program for her neurologic deficits, with relevant improvement. In spite of the good response obtained through second-line therapy, the splenomegaly persisted, and later the gall bladder was surgically removed because of acute cholecystitis (**Figure 1**).

**Table 1:** Patient’s clinical investigations at onset.

	PT	N.R.
***Complete blood count***
**Hemoglobin** (g/dl)	8.7	(11.8 – 14.8)
**WBCs** (cell/mmc)	7140	(4100 – 12000)
**Platelets** (cell/mmc)	352000	(190000 – 460000)
**Lymphocytes** (%)	27.9	(20 – 60)
**Neutrophils** (%)	39.3	(35 –70)
**Monocytes** (%)	13.2	(2 – 12)
**Eosinophils** (%)	18.2	(0 – 6)
**Basophils** (%)	1.4	(0 – 3)
**Reticulocytes** [×10^9^ cell/l (%)]	214 (11.9)	22.5 - 110 (0.5 - 2.5)
**Direct Coombs test**	Strong positive (+++)	Neg.
**Ferritin** (ng/ml)	150	(15 – 300)
**Vitamin B12** (pg/ml)	532	(160 – 800)
**IgG** (mg/dl)	451	(650 – 1500)
**IgA** (mg/dl)	40	(50 – 240)
**IgM** (mg/dl)	103	(50 – 180)
**Bone marrow aspirate**	Increased cellularity with normal trilineage hematopoiesis	
***Vaccination responses***		
**Rubella IgG** (kU/l)	9	> 8
**Measles IgG**	Neg.	Pos.
**Mumps IgG**	Neg.	Pos.
**PCR EBV**	Neg.	Neg.
**PCR CMV**	Neg.	Neg.
**Intradermal tubercolin test**	Neg.	Neg.
***Serology***		
**HIV**	Neg.	Neg.
**EBV**	Neg.	Neg.
**CMV**	Neg.	Neg.
**HCV**	Neg.	Neg.
**Adenovirus**	Neg.	Neg.
**Bartonella hensleae and quintana**	Neg.	Neg.
**Leishmania**	Neg.	Neg.
**Toxoplasma**	Neg.	Neg.
***Lymphocyte count and percentage***		
**T cells** [cell/µl (% of lymphocytes)]	467.4 (38.4)	770 – 4000 (55 – 80)
**CD4 T cells** [cell/µl (% of lymphocytes)]	274.4 (22.6)	400 – 2500 (30 – 50)
**CD8 T cells** [cell/µl (% of lymphocytes)]	138.7 (11.4)	200 – 1700 (14 – 38)
**DNT cells** [cell/µl (% of CD3+ T cells)]	46 (3.8)	(< 2)
**B cells** [cell/µl (% of lymphocytes)]	654.2 (53.8)	100 – 800 (6 – 30)
**NK cells** [cell/µl (% of lymphocytes)]	94.8 (7.8)	70 – 590 (5 – 25)
**Serum IL-10** (pg/ml)	5.9	1
**Serum IL-17A** (pg/ml)	2.6	0.4

N.R., normal range; PT, patient; H.C., healthy control; DNT, double negative T cells.

## Diagnostic Assessment and Treatment

In light of the patient’s clinical presentation, laboratory findings, and poor response to first- and second-line treatments of AIHA, the patient was suspected of having a Primary Immune Regulatory Disorder. Given this suspicion, whole exome sequencing (WES) for immune dysregulation-associated genes analyses were performed, revealing a *de novo* heterozygous germline *STAT3* mutation (c.2144C>T, p.P715L), previously reported as a GOF mutation (8). Cytokine analysis showed increased IL-17A and IL-10 serum levels (**Table 1**). In parallel with the molecular testing, we performed a blood cell morphology analysis which revealed the presence of spherocytes and stomatocytes, and red blood cell fragility tests, along with EMA-binding and protein electrophoresis, confirmed the concomitant diagnosis of hereditary spherocytosis due to spectrin deficiency.

*STAT3* GOF patients are usually treated with immunomodulatory drugs, although targeted therapies interfering with upstream regulators of STAT3 activity (such as IL-6–receptor inhibitors and JAK inhibitors) are currently being evaluated. Considering the severity of the patient clinical condition, probably worsened by associated spherocytosis, we proceeded with hematopoietic stem cell transplantation (HSCT) from a matched unrelated donor (10/10 match), after reduced intensity conditioning including treosulfan, thiotepa, and fludarabine. Cyclosporine, anti-thymocyte globulin (ATG) and methotrexate were used as graft-versus-host disease (GvHD) prophylaxis (**Table S1a**). Engraftment occurred at day 35. The post-transplant course was complicated by low-grade intestinal and skin GvHD, which was however successfully treated with steroids. Cyclosporine dosing was progressively increased in order to achieve the therapeutic range. Nevertheless, at day 49 the patient developed signs of cyclosporine toxicity with papilledema, small retinal hemorrhages, with improvement after stopping cyclosporine.

**Figure 2:**
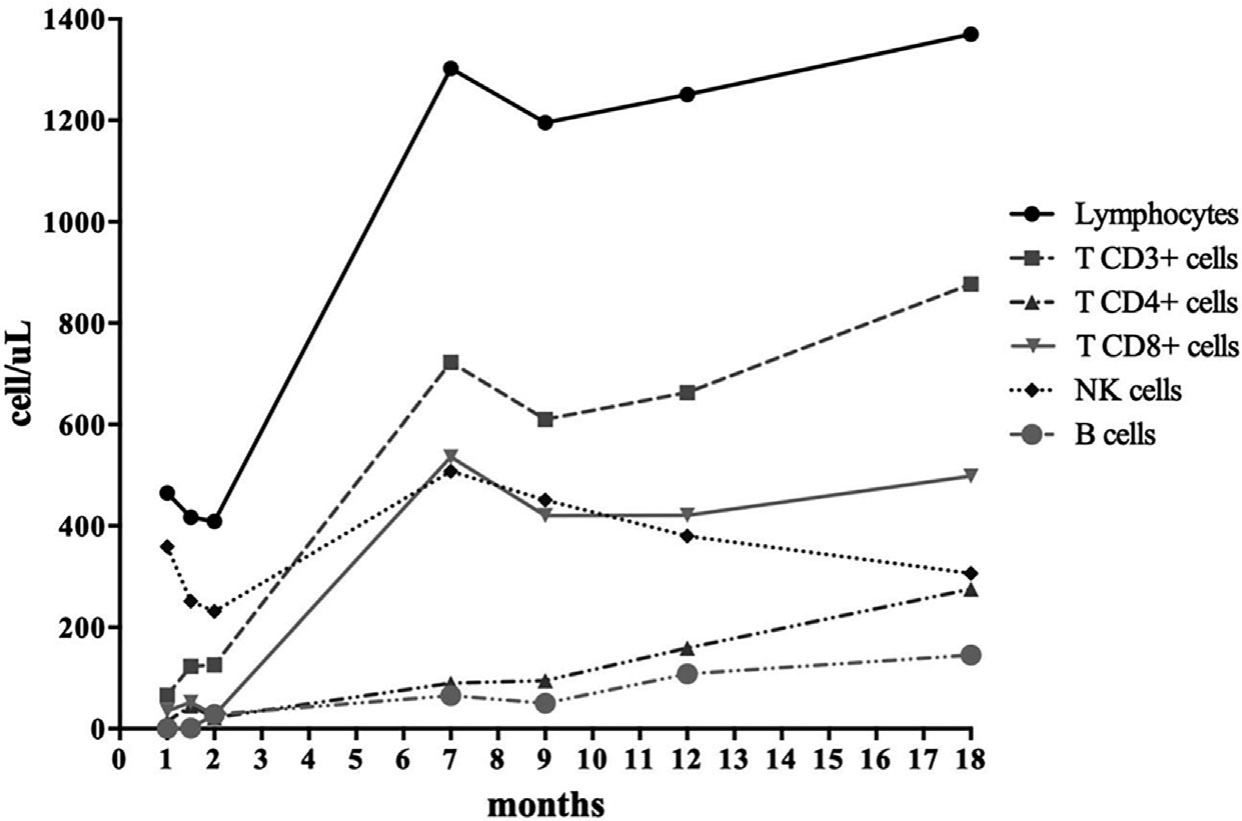
Patient’s immune reconstitution: T, B, and NK cell populations.

Further investigations on cyclosporine toxicity revealed a homozygous SNP of CYP3A4*18B allele, linked to enhanced CYP3A4 activity and to a rapid cyclosporine metabolism. This result is consistent with the low cyclosporine serum levels identified despite the high dose administered, thus leading to the hypothesis that the neurotoxic adverse effects were not due to the accumulation of cyclosporine itself but more likely of its metabolites (9, 10). Overall, the patient achieved full donor engraftment and good immune reconstitution over 18 months post-HSCT (**Figure 2** and **Table S1b**), only requiring regular immunoglobulin infusions. She remained in good clinical conditions without any major complications. However, she suffers from growth delay currently under investigations. Notably, growth defects were recently reported as a predominant feature in this condition (11).

## Discussion

To our knowledge, this is the first case report of a patient with severe refractory hemolytic anemia due to *STAT3* GOF mutation associated with spectrin deficiency. Patients with *STAT3* GOF mutations can have very heterogeneous clinical and immunological disease features (12). Most patients present within the first year of life with lymphoproliferation, autoimmune organ disease (especially endocrine disease), autoimmune cytopenias and growth impairment (8). The *STAT3* P715L mutation we identified has been associated with enhanced transcriptional activation, according to luciferase reporter assays previously conducted (12, 13). Moreover, rapid P715L STAT3 phosphorylation upon IL-6 stimulation - as well as intracellular accumulation of pSTAT3 in the nucleus and cytoplasm - was also previously observed (13). In spite of the hyperactivated profile associated with the mutation, our case can be considered an atypical presentation of *STAT3* GOF, as suggested by delayed onset, mild lymphoproliferation, hypogammaglobulinemia and cytopenia evident as isolated hemolytic anemia. Notably, even though splenomegaly, lymphoproliferation and cytopenia were described in most patients with this condition, hypogammaglobulinemia was not a predominant feature (8). Additionally, our patient displayed highly positive direct Coombs test, but no other autoimmune markers were found. We can hypothesize that our patient’s conditions might have been further complicated by spectrin deficiency, explaining the poor response to immune suppression. Interestingly, in the era of next-generation sequencing, co-occurrence of genetic diseases has been observed (14) and can support unique clinical phenotypes. Even though it is particularly evident in consanguineous pedigrees, this hypothesis cannot not be excluded for autosomal dominant diseases in patients from non-consanguineous parents. The presence of two clinical condition with overlapping features needs to be taken into account for a proper diagnosis and treatment.

Severe anemia as presenting symptom in patients with the same mutation in *STAT3* was also recently reported by Mauracher et al. (13). Rather than peripheral destruction, they indicated a red blood cell production defect by demonstrating suppression of erythropoiesis. The suppression was attributed to increased phosphorylation of STAT3 in patients with P715L *STAT3* mutation, which hinders the required increase in pSTAT5b to respond to elevated erythropoietin levels. This imbalance was evident by the low erythropoietic potential of peripheral CD34^-^ precursor cells, in addition to bone marrow infiltration by oligoclonal lymphoid cells (13). However, in our case bone marrow evaluation showed a normal trilineage hematopoiesis (**Table 1**), allowing us to exclude a lineage defect as responsible for our patient’s clinical conditions.

Taking into account that immunosuppression and targeted therapies represent the gold standard treatment for patients harboring *STAT3* GOF mutations, the definitive and recommended cure for these conditions is HSCT, as shown in our case (**Figure 2** and **Table S1b**).

Since HSCT reverses most of the *STAT3* GOF clinical phenotypes with good long-term outcomes, early-onset autoimmunity should be considered a warning sign of PIRD in order to achieve a prompt diagnosis and appropriate treatment. However, clinical presentation can be occasionally distorted by concomitant medical issues, and it represents a paramount example of how clinical practice should be supported by timely therapeutic intervention.

## Data Availability Statement

The raw data supporting the conclusions of this article will be made available by the authors, without undue reservation.

## Ethics Statements

The studies involving human participants were reviewed and approved by Pediatric Ethics Committee, Meyer University Children’s Hospital. Written informed consent to participate in this study was provided by the participants’ legal guardian/next of kin. Written informed consent was obtained from the minor(s)’ legal guardian/next of kin for the publication of any potentially identifiable images or data included in this article.

## Author Contributions

SC and ES performed immunophenotyping analysis. MC and MV performed genetic analysis. CL and EB performed SNP genotyping. AG and MD’E performed cytokine analysis. EG, CF, AA, IF, AT, VT, SF, and MS supplied patient care. SC, RG, and ES collected and analyzed data. SC, RG, ES, and EG wrote the original draft of the article. All authors contributed to the article and approved the submitted version.

## Funding

This work was supported by the Ministry of Health grant (Ricerca Finalizzata 2016, Ministero Della Salute RF-2016-02362384) and by the Jeffrey Modell Foundation Specific Defect Research Grant (EG); RG was funded by the European Society for Immunodeficiencies (ESID) Medium-term fellowship 2019.

## Conflict of Interest

The authors declare that the research was conducted in the absence of any commercial or financial relationships that could be construed as a potential conflict of interest.

## Supplementary Material

Supplementary Table 1
**(a)** Patient’s conditioning regimen and graft-versus-host disease (GvHD) prophylaxis medications. **(b)** Complete blood count, immunoglobulin levels, vaccination responses, lymphocyte count and percentage of lymphocyte subsets 18 months post-HSCT. N.R., normal range; PT, patient; H.C., healthy control; DNT, double negative T cells; TEMRA, terminally differentiated effector memory T cells; CM, central memory T cells; EM, effector memory T cells; RTE, recent thymic emigrants cells; MZ-like, marginal zone-like B cells.

Supplementary Table 2 Gene panel analysed by NGS.
