# Supplementary material for: Case Report: Signal Transducer and Activator of Transcription 3 Gain-of-Function and Spectrin Deficiency: A Life-Threatening Case of Severe Hemolytic Anemia
Source: Front Immunol. 2021 Jan 15;11:620046. doi: 10.3389/fimmu.2020.620046 (PMC7843414; doi:10.3389/fimmu.2020.620046)
Supplement: Supplementary Table 1 — (a) Patient’s conditioning regimen and graft-versus-host disease (GvHD) prophylaxis medications. (b) Complete blood count, immunoglobulin levels, vaccination responses, lymphocyte count and percentage of lymphocyte subsets 18 months post-HSCT. N.R., normal range; PT, patient; H.C., healthy control; DNT, double negative T cells; TEMRA, terminally differentiated effector memory T cells; CM, central memory T cells; EM, effector memory T cells; RTE, recent thymic emigrants cells; MZ-like, marginal zone-like B cells. [file Table_1.docx]

Supplementary Data

**Table S1. (a)** Patient’s conditioning regimen and graft-versus-host disease (GvHD) prophylaxis medications. **(b)** Complete blood count, immunoglobulin levels, vaccination responses, lymphocyte count and percentage of lymphocyte subsets 18 months post-HSCT. N.R., normal range; PT, patient; H.C., healthy control; DNT, double negative T cells; TEMRA, terminally differentiated effector memory T cells; CM, central memory T cells; EM, effector memory T cells; RTE, recent thymic emigrants cells; MZ-like, marginal zone-like B cells.

**Table S1a.**

| **Conditioning: reduced intensity conditioning** | |  |
| --- | --- | --- |
| *Medication* | *Dose* | *Duration* |
| **Treosulfan** | 14 g/mq/day | 3 days (-6, -5, -4) |
| **Fludarabine** | 40 mg/mq/day | 4 days (-6, -5, -4, -3) |
| **Thiotepa** | 10 mg/kg/dose (2 doses/day) | 1 day (-7) |
| **GvHD prophylaxis** |  |  |
| *Medication* | *Dose* | *Duration* |
| **Anti-thymocyte globulin (ATG)** | 10 mg/kg | 3 days (-5, -4, -3) |
| **Methotrexate** | 15 mg/mq/day – Starting/loading dose | 1 day (+1) |
|  | 10 mg/mq/day – Maintenance dose | 3 days (+3, +6, +11) |
| **Cyclosporine** | 2 mg/kg/day infusion IV | Started on day -2 |
|  |  | (Aimed for drug level: 150-200 ng/ml) |
|  |  | Administered for 6 weeks |

**Table S1b.**

|  | ***PT*** | ***N.R.*** |
| --- | --- | --- |
| ***Complete blood count*** |  |  |
| **Hemoglobin** (g/dl) | 14.7 | (11.8 – 14.8) |
| **WBCs** (cell/mmc) | 4790 | (4100 – 12000) |
| **Platelets** (cell/mmc) | 151000 | (190000 – 460000) |
| **Lymphocytes** (%) | 28.6 | (20 – 60) |
| **Neutrophils** (%) | 56.5 | (35 –70) |
| **Monocytes** (%) | 7.0 | (2 – 12) |
| **Eosinophils** (%) | 4.7 | (0 – 6) |
| **Basophils** (%) | 0.8 | (0 – 3) |
| **IgG** (mg/dl) | 585 | (650 – 1500) |
| **IgA** (mg/dl) | 42.5 | (50 – 240) |
| **IgM** (mg/dl) | 34 | (50 – 180) |
| ***Vaccination responses*** |  |  |
| **Hepatitis B antibody (HBsAb)** (mUI/ml) | 57.3 | > 10 |
| **Tetanus** (UI/ml) | > 1 | (Protective) |
| **Diphtheria** (UI/ml) | > 1 | (Protective) |
| ***Lymphocyte count*** |  |  |
| **Lymphocytes** (cell/µL) | 1370 | 2400 (1200 – 4700) |
| **CD3 T** (cell/µL) | 877 | 1800 (770 – 4000) |
| **CD4 T** (cell/µL) | 275 | 1000 (400 – 2500) |
| **CD8 T** (cell/µL) | 498 | 600 (200 – 1700) |
| **NK** (cell/µL) | 306 | 200 (70 – 590) |
| **B** (cell/µL) | 145 | 290 (100 – 800) |
| ***T cell subpopulations*** |  |  |
| **HLA-DR Activated T cells** (% lymphocytes) | 3.3 | 7 (3 – 14) |
| **DNT cells** (% CD3+ T cells) | 1.4 | < 2 |
| **Naïve CD4 T cells** (% CD4+ cells) | 66.8 | 67 (46 – 99) |
| **CM CD4 T cells** (% CD4+ cells) | 30.3 | 18 (0.35 – 100) |
| **EM CD4 T cells** (% CD4+ cells) | 2.8 | 2 (0.27 – 18) |
| **TEMRA CD4 T cells** (% CD4+ cells) | 0.1 | 0.1 (0.0031 – 1.8) |
| **RTE CD4 cells** (% CD4+ cells) | 50.2 | 58 (41 – 81) |
| **Treg** (% CD4+ cells) | 2.9 | 8 (4 – 14) |
| **Naïve CD8 T cells** (% CD8+ cells) | 48.5 | 75.8 (57 – 83.7) |
| **CM CD8 T cells** (% CD8+ cells) | 34.9 | 16.6 (9.2 – 22.6) |
| **EM CD8 T cells** (% CD8+ cells) | 6.3 | 2.3 (0.7 – 14) |
| **TEMRA CD8 T cells** (% CD8+ cells) | 10.4 | 3.8 (0.9 – 17.9) |
| ***B cell subpopulations*** |  |  |
| **Naïve B cells** (% CD19+ cells) | 85.3 | 75.4 (69.4 – 80.4) |
| **Class switch B cells** (% CD19+ cells) | 7.2 | 6.5 (5.2 – 12.1) |
| **MZ-like B cells** (% CD19+ cells) | 3.3 | 10 (7.5 – 12.4) |
