# Supplementary material for: Case Report: Signal Transducer and Activator of Transcription 3 Gain-of-Function and Spectrin Deficiency: A Life-Threatening Case of Severe Hemolytic Anemia
Source: Front Immunol. 2021 Jan 15;11:620046. doi: 10.3389/fimmu.2020.620046 (PMC7843414; doi:10.3389/fimmu.2020.620046)
Supplement: Supplementary Table 2 — Gene panel analysed by NGS. [file Table_2.docx]

Supplementary Data

**Table S2.** Gene panel analysed by NGS

| **GENE** | **GenBank N° (GRCh38.p7/hg38)** | **OMIM** |
| --- | --- | --- |
| *ADA* | NM_000022.3 | 608958 |
| *AIRE* | NM_000383.3 | 607358 |
| *BCL10* | NM_003921.4 | 603517 |
| *BCL6* | NM_001706.4 | 109565 |
| *CARD11* | NM_032415.5 | 607210 |
| *CASP10* | NM_032977.3 | 601762 |
| *CASP8* | NM_001228.4 | 601763 |
| *CD27* | NM_001242.4 | 186711 |
| *CD70* | NM_001330332.1 | 602840 |
| *CECR1 (ADA2)* | NM_001282225.1 | 607575 |
| *CORO1A* | NM_007074.3 | 605000 |
| *CTLA4* | NM_005214.4 | 123890 |
| *CTPS1* | NM_001905.3 | 123860 |
| *CTPS2* | NM_019857.4 | 300380 |
| *CXCR4* | NM_003467.2 | 162643 |
| *DOCK2* | NM_004946.2 | 603122 |
| *DOCK8* | NM_203447.3 | 611432 |
| *FAS* | NM_000043.5 | 134637 |
| *FASLG* | NM_000639.2 | 134638 |
| *FCGR3A* | NM_001127593.1 | 146740 |
| *FOXP3* | NM_014009.3 | 300292 |
| *GATA2* | NM_032638.4 | 137295 |
| *IKBKG (NEMO)* | NM_001099856.4 | 300248 |
| *IL10* | NM_000572.2 | 124092 |
| *IL10RA* | NM_001558.3 | 146933 |
| *IL10RB* | NM_000628.4 | 123889 |
| *IL2RA* | NM_000417.2 | 147730 |
| *ITCH* | NM_001257137.2 | 606409 |
| *JAK2* | NM_004972.3 | 147796 |
| *JAK3* | NM_000215.3 | 600173 |
| *KLHDC8B* | NM_173546.2 | 613169 |
| *LRBA* | NM_006726.4 | 606453 |
| *MAGT1* | NM_032121.5 | 300715 |
| *MALT1* | NM_006785.3 | 604860 |
| *MCM4* | NM_005914.3 | 602638 |
| *NFKBIA* | NM_020529.2 | 164008 |
| *NLRC4* | NM_021209.4 | 606831 |
| *ORAI1* | NM_032790.3 | 610277 |
| *PGM3* | NM_001199917.1 | 172100 |
| *PIK3CD* | NM_005026.3 | 602839 |
| *PIK3R1* | NM_181523.2 | 171833 |
| *PIK3R5* | NM_014308.3 | 611317 |
| *PLCG2* | NM_002661.4 | 600220 |
| *PLCG2* | NM_002661.4 | 600220 |
| *PRKCD* | NM_006254.3 | 176977 |
| *RAG1* | NM_000448.2 | 179615 |
| *RAG2* | NM_000536.3 | 179616 |
| *RASGRP1* | NM_005739.3 | 603962 |
| *STAT1* | NM_007315.3 | 600555 |
| *STAT3* | NM_139276.2 | 102582 |
| *STAT4* | NM_003151.3 | 600558 |
| *STAT5A* | NM_003152.3 | 601511 |
| *STAT5B* | NM_012448.3 | 604260 |
| *STIM1* | NM_003156.3 | 605921 |
| *STK4* | NM_006282.3 | 604965 |
| *TET2* | NM_001127208.2 | 612839 |
| *TMEM173 (STING)* | NM_198282.3 | 612374 |
| *WAS* | NM_000377.2 | 300392 |
| *ZAP70* | NM_001079.3 | 176947 |
